# Oviposition Deterrent Efficacy and Characteristics of a Botanical Natural Product,* Ocimum gratissimum* (L.) Oil-Alginate Beads, against* Aedes aegypti* (L.)

**DOI:** 10.1155/2018/3127214

**Published:** 2018-08-01

**Authors:** Saraporn Harikarnpakdee, Chomnapas Chuchote

**Affiliations:** ^1^Department of Industrial Pharmacy, Faculty of Pharmacy, Rangsit University, Pathumthani 12000, Thailand; ^2^Department of Pharmacognosy, Faculty of Pharmacy, Rangsit University, Pathumthani 12000, Thailand

## Abstract

This study was aimed at investigating the oviposition deterrent activity of* Ocimum gratissimum* (L.) essential oil (*O. gratissimum *oil) and its product,* Ocimum gratissimum* (L.)- alginate beads (beads), against* Aedes aegypti* (*Ae. aegypti*) mosquitoes. Chemical analysis of* O. gratissimum* oil obtained by hydrodistillation, using gas chromatography-mass spectroscopy techniques, presented eugenol (67.38%) and Z-*β*-ocimene (14.95 %) as major constituents. Good characteristics of beads were obtained by the orifice-ionic gelation method with calcium chloride as hardening agent and Tween®20 as emulsifier. The beads exhibited a good spherical shape and good hardness and flexibility with an average size of 1.49 ± 1.36 mm. The oil content, the yield percentage, and the entrapping efficiency were also examined. The beads (formulation code, F2) could prolong the essential oil release until the 10^th^ d. This beads provided a remarkably longer oviposition deterrence activity against gravid* Ae. aegypti* with high percentage for 27 d, whereas free* O. gratissimum* oil showed a short period of time (8 d) in this activity. The stability study showed the stability of oil content and its compositions in storage condition. These results are very affordable approaches to control the dengue fever.

## 1. Introduction

Dengue is one of the most prevalent pandemic threats caused by mosquitoes. More than 100 countries with 75% of the global population are facing domestic mosquito-borne viral diseases, especially in the Asia-Pacific region. Thousands of deaths globally reported each year were caused by the most typical vector of dengue,* Ae. aegypti* [1]. One of the most preferential breeding habitats was found to be in the human living sheltered places [2]. In order to control the spreading of the disease caused by dengue virus, synthetic insecticide-based control strategies including organochlorines, carbamates, pyrethroids, and larvicide were used [3]. However, the success rate is obstructed due to the development of resistance strands in the virus [4, 5]. Moreover, vaccine-based control strategies were also investigated, aiming to reduce the prevalent rate of the disease. Despite 50 years of research, there still have been no promising vaccines available up till now [6, 7]. Other alternative strategies have also been explored, but none of them have been successful. It is indeed necessary to find an effective, environmentally safe, biodegradable, low cost, practical strategy to control the spreadability of the mosquitoes-based diseases. Recent mosquito repellent research had focused on the potential use of* O. gratissimum *oil, a kind of oil naturally produced from an indigenous perennial scented shrub. It was highly evidenced that this plant, the Lamiaceae family, possesses a biological control of mosquito-borne diseases [8–10]. Traditionally, the essential oil extracted from* O. gratissimum* leaves has been reported to have repellent effects and larvicidal and insecticidal activities [11–13].* O. gratissimum* was long used to control the transmissibility of* Ae. Aegypti* [14–17]. However, the information of the mosquito oviposition deterrent activity of* O. gratissimum* is still limited. In addition, the high volatility and degradation rates of* O. gratissimum *oil when exposed to high temperatures, oxidants, and UV radiation also hindered the biological activity of the essential oil. Beads are a type of polymer formed into a small container wrapping around the active components in order to prevent the exposure of the bioactive compound to the environment and also to harden the liquid formulation of the ease of transportation [18]. Alginate beads have been introduced in the food and pharmaceutical industries to increase the bioavailability of the active component. A sodium salt of alginic acid or sodium alginate is able to form a cross-linked insoluble structure with valent cations by the orifice-ionic gelation method which results in a large and uniform shape of the beads [19, 20]. The alginate bead system allows the active components to slowly diffuse through the wall in a control-released manner. This system will also enhance the efficacy, stability, and suitability of the pesticide active component [21]. Additionally, the natural polymer-based system is also biodegradable and biocompatible providing a nonimmunogenic approach for the drug delivery system [22, 23].

This study prepared and reported the characteristics of* O. gratissimum *oil entrapped in biopolymer beads by the orifice-ionic gelation method including the oil content, the yield percentage, and the entrapping efficiency. The bead system was also examined for an overall morphology, a drug release effect, and stability. The efficacy of the system to inhibit oviposition was confirmed by the* Ae. aegypti* bioassay model.

## 2. Materials and Methods

*Materials*. Sodium alginate was purchased from Sigma Aldrich (St. Louis, USA). Polyoxyethylene sorbitan monolaurate (Tween 20) was obtained as a kind gift from Tariko Co., Ltd. (Bangkok, Thailand). Calcium chloride was purchased from Merck (Darmstadt, Germany). Essential oil was obtained from* O*.* gratissimum* plant as per description in the extraction section.

*Extraction of Essential Oil and Oil Yield*. The aerial parts of* O. gratissimum* were gathered from a farm in Pathumthani during the balmy period and distilled within 24 h by hydrodistillation using a Clevenger-type apparatus. About 1-2 kg of fresh leaves were cut into small pieces and packed in a distillation flask. A sufficient quantity of water was added and brought to a boil. Live steam is alternatively injected into the plant charge. The distillation time was about 4 h, and the vapor mixture of water and oil was condensed by indirect cooling with water. The obtained oil was automatically separated from the distillate water into a separator.* O. gratissimum* oil was dried over anhydrous sodium sulphate and kept in a dark screwed-cap glass vial at a temperature of 4°C until used. The yield of* O. gratissimum* oil was recorded, and the reported values were the mean of at least 3 distillations [24].

*Analysis of Chemical Constituents*.* O. gratissimum* oil was analysed for chemical constituents, employing the gas chromatography-mass spectroscopy (GC-MS) assay. Briefly, the essential oil (20 *μ*l) was diluted with 2 ml methanol to a final concentration of 1% (v/v). The diluted sample (1 *μ*l) was then injected into a BPx5 fused silica capillary column (30 m x 0.25 mm x 0.25 *μ*m) for analysis with a GC-MS instrument (Varian Saturn 3). The operation conditions were as follows: the injection temperature was 180°C; helium was used as a carrier gas; the purge flow rate was 1 ml/min; and the split ratio was 1:100. The chemical constituents of essential oil were identified by matching their mass spectra and retention indices with Adams EO Mass Spectral library and the National Institute of Science and Technology (NIST). Mass Spectral library and the percentage composition were computed from GC peak areas.

*Preparation of Beads*.* O. gratissimum *oil-alginate beads were prepared, solidifying them in liquid (orifice-ionic gelation method) [25]. The bead formulations as presented in Table 1 consisted of a variety of amounts of* O. gratissimum *oil prepared with or without Tween 20 as an emulsifier to obtain proper characteristics of beads and to study these effects on formulations. Sodium alginate was dissolved in distilled water to form 1% (w/w) of homogeneous polymer solution. The* O. gratissimum* oil with or without Tween 20 (1% w/w) was added to the polymer solution in a concentration of 0.25-60% (w/w) and mixed thoroughly under continuous mechanical stirring for 10 min in a 1000 rpm speed at a room temperature to make a viscous emulsion. The resulting emulsion was then added manually dropwise into a hardening bath containing 250 ml of 2% (w/v) of calcium chloride solution as a hardening agent through a syringe with a needle (size no. 24) at a height of 5 cm above the surface of the hardening solution. The added droplets were retained in the stagnant calcium chloride solution for 15 min to complete the curing reaction and to produce spherical rigid beads, followed by washing twice with distilled water. The beads were filtered and dried at a room temperature for 12 h to evaporate water on the bead surface [26].

*Essential Oil Content, Entrapping Efficiency, and Production Yield*. The* O. gratissimum* oil content was determined using the modified method described in previous publication [24]. Accurately weighed amount of beads (*W*) was dispersed in distilled water and then sonicated about 10 min until the shell of beads was broken. Hydrodistillation with a Clevenger-type apparatus was used for the extraction. The entrapped oil obtained by hydrodistillation (*W*_1_) was weighed, and the essential oil content was calculated in percentage with average of three determinations. The essential oil content was defined as in the following equation:

(1) $$\begin{array}{c} Percentage\;\;\text{of}\;\;essential\;\;oil\;\;content=\left(\;\frac{W_{1}}{W}\;\right)\;x\;100 \end{array}$$

The entrapping efficiency value was an average of three determinations. The actual amount of entrapped* O. gratissimum* oil extracted from beads by hydrodistillation (*W*_1_) relative to the theoretical amount of entrapped* O. gratissimum *oil (*W*_2_) was calculated for entrapping efficiency in percentage using the following formula:

(2) $$\begin{array}{c} Percentage\;\;\text{of}\;\;entrapping\;\;efficiency=\left(\;\frac{W_{1}}{W_{2}}\;\right)\;x\;100 \end{array}$$

The percentage of production yield was calculated based on the weight of beads (*W*_3_) recovered from the preparation and the sum of the initial dry weights of starting materials (*W*_4_) comprising* O. gratissimum* oil, sodium alginate, and Tween 20 as shown in (3). The reported values are the means of three determinations.

(3) $$\begin{array}{c} Percentage\;\;\text{of}\;\;production\;\;yield=\left(\;\frac{W_{3}}{W_{4}}\;\right)\;x\;100 \end{array}$$

*Particle Size Measurement and Morphological Examination*. The mean size of the beads was determined by an optical microscope (BA-300, Motic Corporation, Germany) connected with Axiovision Rel. 4.8 software (Zeiss Standard Universal, Zeiss, Germany) from the average of 100 particles. The morphology and the microstructure of beads were examined by optical microscopy (BA-300, Motic Corporation, Germany).

*Chemical Constituents of Entrapped Oil*. The entrapped oil was extracted by hydrodistillation, as previously mentioned, and was determined for chemical constituents by GC-MS analyses.

*In Vitro Release Study*. The release profiles of free* O. gratissimum *oil and entrapped* O. gratissimum* oil were investigated by the slightly modified method under laboratory conditions at room temperature [24]. The beads with a dosage equivalent to 0.5 g of essential oil content were placed in black plastic cups (250 ml in capacity) filled with 200 ml stagnant distilled water and covered with nylon nets. At time intervals (0, 1, 2, 3, 4, 5, 6, 7, 8, 9, and 10 d), each sample was analysed for the residual content of entrapped essential oil by using eugenol (major constituent) as the chromatographic tracer of the* O. gratissimum* oil in the HPLC system. Each sample obtained at those time intervals was filtered through filter paper and dispersed in 20 ml of methanol in a 50-ml polypropylene tube. The tube was sonicated about 10 min until the shell of beads was broken. The dispersion was filtered through a 0.45 um-PTFE syringe filter. The filtrate was appropriately diluted with methanol and analysed for the residual content of entrapped oil in the sample beads using the estimation by calibration curve [27]. The amount of* O. gratissimum* oil released was obtained by the difference between the initial content and the residual content of entrapped oil in sample beads after release study. The release profile of free* O. gratissimum* oil was also studied in similar conditions. The results obtained were averages of three determinations, and they were reported as percentage release profile.

*Construction of Calibration Curve*. A stock solution of 2 mg/ml of eugenol standard solution was prepared in methanol. Working solutions were prepared by diluting the stock solution with the same solvent to obtain 5, 10, 25, 50, 75, and 100 ug*/*ml. Each concentration was prepared with three replicates and injected into the HPLC system. The amount of* O. gratissimum* oil corresponding to the eugenol content was calculated by using eugenol peak area and the concentration of eugenol based on the calibration curve.

*HPLC Condition*. Eugenol was analysed by the HPLC system using an Agilent 1260 infinity system equipped with a photodiode array detector (Agilent Technologies, USA). Separation was achieved at 25°C on a ZORBAX Eclipse Plus C18 (100 x 4.6 mm, i.d., 3.5 um). The mobile phase consisted of water (40% v/v) and methanol (60% v/v) with a flow rate of 1 ml/min. The injection volume was 10 ul. The quantitation wavelength was set at 280 nm. The HPLC validated method had a good linearity (R^2^ = 0.9994) at a concentration ranging from 5 to 100 ug/ml. The limits of detection and quantitation were 25 ng/ml and 62.5 ng/ml, respectively. This method showed a good precision, and the relative standard deviation was less than 0.6% for both intraday and interday precision. In addition, the accuracy represented as percent recovery was 96.17-103.90%. However, the method validation was described in previous publication [28].

*Oviposition Deterrent Test*. To investigate oviposition deterrent activity, the slightly modified method described in previous publication [29, 30] was used. This study used gravid female* Ae. aegypti* mosquitoes aged 4-5 days obtained from the Division of Entomology, National Institute of Health of Thailand, Department of Medical Sciences, Thailand. They were placed in an oviposition cage. The oviposition deterrent activity of different amounts of* O. gratissimum* oil against gravid* Ae. aegypti* mosquitoes under laboratory conditions at a room temperature was studied. Two black plastic cups (250 ml in capacity) were placed in the oviposition cage. One cup was filled with 200 ml of distilled water as a control and the other was treated with empty beads (1 g) and distilled water (200 ml) or* O. gratissimum *oil at different amounts of 0.1, 0.3, 0.5, and 0.7 g mixed with distilled water with final volume of 200 ml. The final concentrations of the treated essential oil in each treated cup were 0.5, 1.5, 2.5, and 3.5 mg/ml, respectively. Each cup was fitted inside with a white filter paper sheet (7 x 28 cm^2^) for the deposition of mosquito eggs. The paper was located in each cup so that the lower half of the paper was submerged in distilled water. The control and treated cups were placed at diagonally opposite corners in a mosquito cage (30 x 30 x 30 cm^3^) containing 20 gravid female mosquitoes for 24 h. Then, the eggs laid in each cup were counted after the removal of the oviposition paper. Six replicates were made in each case. The percentage of deterrence for each test was calculated by applying the following equation [30].

(4) $$\begin{array}{c} Percentage\;\;\text{of}\;\;deterrence=\frac{\left(C–T\right)}{C}\;x\;100 \end{array}$$

*C* stands for the number of mosquito eggs collected from the control cup and* T* denotes the number of mosquito eggs collected from the treated cup.

*Persistency of Oviposition Deterrent Activity*. The persistency of the oviposition deterrent effect after application of free* O. gratissimum *oil and* O. gratissimum *oil-alginate beads was investigated. For this investigation,* O. gratissimum* oil was applied at the lowest dosage yielding 100% deterrence of 20 gravid female mosquitoes within 24 h (2.5 mg/ml; 0.5 g of essential oil in 200 ml of distilled water), and beads (F2) were applied at dosage equivalent to 0.5 g of essential oil content. The persistency of oviposition deterrent activity against gravid* Ae. aegypti *was studied under laboratory conditions at a room temperature as previously described [29, 30]. The experiment was performed in six replicates. Thus, the total number of gravid* Ae. aegypti* was 120 for each treatment, and the percentage of deterrence was determined after 24 h of exposure. The eggs laid in the control and treated cups were counted after the removal of the oviposition paper, and the new filter paper sheets were fitted inside the control and treated cups which, then, were placed in a new mosquito cage with a new batch of 20 gravid female* Ae. Aegypti.* The percentage of deterrence was counted 24 h after placement. The treatment was challenged daily with a fresh batch of 20 gravid* Ae. aegypti*. The assays were performed continuously until the percentage of deterrence in each treated group reached zero. The results of the experiments were reported as the average of individual treatment.

*Storage Stability*.* O. gratissimum *oil content and chemical constituents of entrapped essential oil were determined by hydrodistillation and GC-MS assay after an exposure of the sample beads in sealed glass containers in an oven at 40°C for 6 weeks. This was assessed as reported in the literature [31].

*Data Analysis*. The percentages of oviposition deterrence against gravid* Ae. Aegypti* among various concentrations of* O. gratissimum* oil were recorded. The recorded percentages were, then, compared using the one-way analysis of variance (ANOVA) with Dunnett's test. All differences were considered to be significant at P < 0.05.

## 3. Results and Discussion

*Yields and Chemical Constituents of O. gratissimum Oil*. The yield of* O. gratissimum *leaf essential oil was 3.54 g/kg fresh weight. Chemical identification of essential oil was accomplished by GC-MS analyses.  Table 2 shows the constituents of the essential oil, their percentage area, and their retention time (min). A total of 16 compounds representing 100 % of the* O. gratissimum* oil were identified. The major constituents of this oil were eugenol (67.38 %) and Z-*β*-ocimene (14.95 %). The percentage compositions of remaining 14 compounds ranged from 0.09 to 5.25 %. The major constituents of this essential oil from leaves of* O. gratissimum* L. reported in this present study were similar to those previously established by Bunrathep et al., 2007 (Pathumthani, Thailand) [32], Matasyoh et al., 2007 (Meru, Kenya) [33], and Malebo et al., 2013 (Dar es Salaam, Tanzania) [13]. In contrast, the essential oil from* O. gratissimum *harvested in Turkey, Cameroon, and Brazil was found to be particularly rich in thymol and *γ*-terpinene [34–36].

*Preparation Process and Characteristics of Beads*. In this experiment, sodium alginate and calcium chloride played the role of shell forming substances and hardening agents, respectively. When droplets of aqueous solution of sodium alginate were added to a calcium chloride solution, an elastic round ball formation of water-insoluble calcium alginate around the* O. gratissimum* oil drop was instantaneously formed. The gelation along with cross-linking of the sodium alginate was achieved by the exchange of sodium ions from the *α*-L guluronic acid with the divalent Ca^2+^ cations and the random *α*-L guluronic groups to form a characteristic egg-box-like structure [38]. In the preliminary study, the bead formulations were prepared without emulsifier (P1-P4). Then, the complete mixing and thorough emulsification of sodium alginate solution and* O. gratissimum *oil could not occur, and the separation between oil phase and polymer solution phase was noticed. It was noticeable that, due to an increase in the amount of essential oil in formulations (P1-P4), the percentage of entrapping efficiency and production yield clearly decreased as shown in Table 1. The low percentage of essential oil content in formulations without emulsifier (P1-P4) was obtained despite more intensified concentration of* O. gratissimum* oil in formulations. These problems were solved by using Tween 20 as an emulsifier in formulations. In order to discuss the optimum formation condition of the beads, the changed factor in preparation was the concentration of* O. gratissimum* oil in 10–60 % (w/w) prepared with Tween 20 as an emulsifier. Table 1 shows that an increase in the amount of* O. gratissimum* oil ranging from 10 to 30% (w/w) with Tween 20 increased the production yield, essential oil content, and entrapping efficiency of the beads. Then, good characteristics of beads were obtained when using Tween 20. This could be explained by the fact that the emulsifier, Tween 20, was used to enhance the solubilization of* O. gratissimum* oil and stabilize the oil droplets in the continuous hydrophilic matrix of the beads [39, 40]. The maximal production yield and the highest entrapping efficiency were obtained from formulations, F2 and F3. At an amount of* O. gratissimum* oil higher than 30% (w/w), these characteristics clearly decreased. It could be explained that when an amount of* O. gratissimum* oil was higher than 30% (w/w), there would obviously be a lot of* O. gratissimum* oil floating on the top layer of the emulsion, and the amount of polymer matrix of the beads was too low to completely cover the* O. gratissimum* oil droplets. The mean particle size of the beads showed no marked differences in all formulations (Table 1).

**Figure 1:**
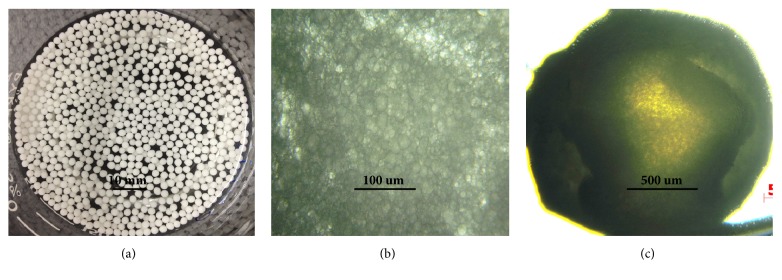
Morphology and microstructure of beads by the optimum condition.

Figure 1 is the microscopic picture of the beads (F2) manufactured under the condition of 20% (w/w) of* O. gratissimum* oil, 1% (w/w) of sodium alginate, and 1% (w/w) of Tween 20. Under this manufacturing condition, a good formation and dispersion of beads could be obtained. As shown in Figure 1(a), the beads exhibited a good spherical shape and good hardness and flexibility with an average size of 1.49 mm.  Figure 1(b) shows that several oil droplets were enclosed within the bead shell.  Figure 1(c) shows that the beads had a nucleus-shell structure with a compact wall.

The methods used in this preparation process did not cause any appreciable modifications in the composition of* O. gratissimum* oil. No marked changes in the oil content and composition were recorded during the stability test, indicating that the entrapped oil was quite stable when stored in an absence of humidity as the stability test condition (Table 2). The mean diameter of the beads before and after the stability test showed no marked differences, indicating that the stability test condition used had no noteworthy influence on the particle size (Table 1).

**Figure 2:**
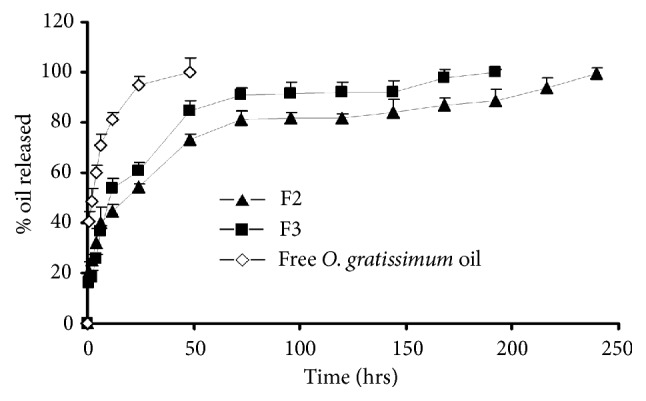
In vitro release profiles of free* O. gratissimum* oil and optimized formulations of alginate beads (F2 and F3).

*Release Profile Study*. Release studies were conducted on two optimized formulations with the maximal production yield and the highest entrapping efficiency (F2 and F3).  Figure 2 shows the* O. gratissimum* oil release profiles of F2 and F3 formulations comparing with release profile of free* O. gratissimum* oil. It was shown that free* O. gratissimum* oil was released up to 88.92% within 24 h, while F2 and F3 formulations only released 54.02% and 60.61%, respectively. This prolongation was explained by the reaction of sodium alginate and calcium chloride. The cross-linking reaction of sodium alginate with calcium chloride is based on the formation of a tight junction between the guluronic acid residues. Ca^2+^ ions, with an atomic size of 1.14 Å, form a two-dimensional bonding structure with sodium alginate inside the alginate matrix causing an increase in the alginate gel density. When the beads were exposed to a moist environment, the beads initially absorbed water. This absorption led to some modifications in the shell material, which became swollen, rubbery, and sticky in appearance leading to the prolongation of the release of essential oil [38]. High burst effect on the first day of release profiles of beads occurred by the cross-linking of essential oil loaded-calcium alginate on the surface of beads, which was rapidly released during the first period of the in vitro release study [39]. Comparing the release profiles of F2 and F3 implies that release rate of beads with low essential oil to alginate mass ratio (F2) is slower than those with high essential oil to alginate mass ratio (F3). Beads of formulations F2 and F3 could prolong the* O. gratissimum* oil release with longer period of 10 and 8 days, respectively. This may be due to the increase in the amount of* O. gratissimum* oil affecting the decrease of the alginate mass and the thickness of alginate wall membrane.

**Figure 3:**
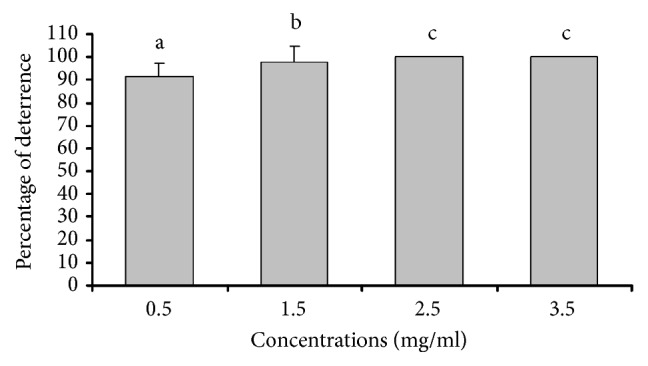
The percentage of oviposition deterrence (mean ± SD) of various* O. gratissimum *oil concentrations. Different letters between columns indicate significant differences (P < 0.05, by one-way ANOVA with Dunnett's test).

*Oviposition Deterrent Activity*. Oviposition deterrent effects with various concentrations (0.5-3.5 mg/ml) of* O. gratissimum* oil solution and empty beads against* Ae. aegypti *are shown in Figure 3. All treated groups exhibited oviposition deterrent activity against* Ae. aegypti *with a high percentage of deterrence ranging from 91.19 to 100 %, whereas empty beads clearly provided no deterrent activity because the mean number of mosquito eggs in the treated group was greater than the control group. The tested concentrations of* O. gratissimum* oil ranging from 0.5 to 2.5 mg/ml showed significant difference (p < 0.05) in oviposition deterrent effects, and the lowest concentration exhibiting 100 % oviposition deterrence was 2.5 mg/ml. According to previous literature,* O. gratissimum* oil with eugenol as a constituent was known to have repellent and larvicidal activity against mosquitoes [13, 14]. Then, in this study, another activity exhibited by* O. gratissimum *oil containing eugenol as a major constituent was oviposition deterrent activity against* Ae. aegypti* with a high percentage of deterrence. In related studies, essential oil from* Alpinia purpurata* with *α*-pinene, *β*-pinene, and *β*-caryophyllene as major constituents at a concentration of 100 ppm was reported to induce a strong oviposition deterrent response against* Ae. Aegypti *[41]. Moreover, a high potential of oviposition deterrent property against* Ae. aegypti* at a concentration of 0.01% (v/v) was observed in various plants including* Curcuma longa*,* Zingiber officinale*,* Vitex trifolia*,* Melaleuca cajuputi*,* Hedychium coronarium*,* Psidium guajava,* and* Houttuynia cordata* [30].

**Figure 4:**
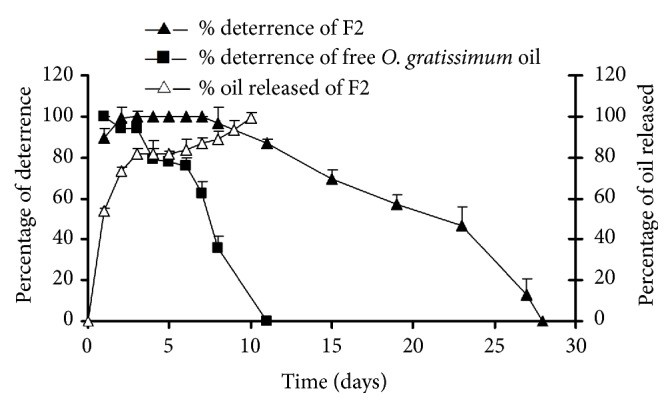
The percentage of oviposition deterrence (mean ± SD) in treatment of* O. gratissimum* oil and* O. gratissimum* oil-alginate beads (F2) and the percentage of oil released from* O. gratissimum* oil-alginate beads (F2).

*Persistency of Oviposition Deterrent Activity*. The persistency study was evaluated using the beads in formulation F2 produced under the maximal yield condition of preparation and the best releasing property of* O. gratissimum *oil. Figure 4 shows the oviposition deterrent efficacy against gravid female* Ae. Aegypti *of free* O. gratissimum* oil and* O. gratissimum *oil-alginate beads. Under laboratory conditions, the fresh preparation of* O. gratissimum* oil-alginate beads used in a dosage equivalent to 0.5 g of essential oil content showed deterrence of more than 80% on the first 2 d and exhibited 100% of deterrence on the 3^rd^ d of the investigation. This beads offered complete oviposition deterrence (100%) for a 5-day period and the deterrence to unacceptable rates (<50%) after 19 d of application. These effects were obviously higher than those of* O. gratissimum *oil alone (0.5 g; the lowest dosage yielding 100% deterrence), which offered a complete oviposition deterrence for only one day, with a decrease in oviposition deterrence to lower than 50% occurring in a shorter period of time (6 d). This indicated that when* O. gratissimum *oil was prepared in a form of alginate beads, it could reserve oviposition activity for a notably longer period of time than that applied in the oil form. A prolonged release of oil from the alginate beads yielded good initial and residual oviposition deterrent efficacy while the* O. gratissimum *oil offered a magnitude of release that was adequate only in the initial period. This effect could be attributed to gradually releasing control of* O. gratissimum* oil from beads by calcium alginate shell (Figure 4) in order to increase a period of time for contact between* Ae. Aegypti *and* O. gratissimum* oil. This could be a reason for the enhanced oviposition deterrent efficacy of* O. gratissimum *oil after the preparation in the form of* O. gratissimum* oil-alginate beads.

## 4. Conclusions

This research used orifice-ionic gelation method to manufacture* O. gratissimum* oil-alginate beads. This method reduces loss of the active principles, leading to high loading beads offering the possibility of controlled release of* O. gratissimum* oil. Beads strongly deter oviposition of* Ae. aegypti* for about 27 d by gradually releasing* O. gratissimum* oil from beads. This action seems attributable to the major constituent as eugenol of phenolic content in this essential oil. These results are very affordable approaches to control the dengue fever.

## Figures and Tables

**Table 1:** Formulations, production yield, essential oil content, entrapping efficiency, and mean diameter of the prepared beads.

Formulation code	Essential oil %(w/w)	Sodium alginate %(w/w)	Tween®20 %(w/w)	Production yield (%)	Essential oil content (%)	Entrapping efficiency (%)	Mean diameter (mm)
P1	0.25	1	—	69.23 ± 4.89	13.90 ± 0.64	48.12 ± 3.25	1.28 ± 2.19
P2	0.5	1	—	66.93 ± 3.47	22.41 ± 0.23	45.00 ± 4.79	1.26 ± 2.47
P3	1	1	—	64.71 ± 3.14	30.13 ± 0.82	39.00 ± 5.13	1.23 ± 3.21
P4	2	1	—	63.29 ± 4.31	27.97 ± 0.92	26.55 ± 3.56	1.10 ± 4.08
F1	10	1	1	69.09 ± 3.56	61.52 ± 0.55	51.00 ± 2.78	1.44 ± 2.52
F2	20	1	1	72.14 ± 4.31	68.36 ± 0.24	54.25 ± 5.14	1.49 ± 1.36
F3	30	1	1	75.00 ± 2.98	83.13 ± 0.52	66.50 ± 3.89	1.54 ± 4.22
F4	40	1	1	63.41 ± 2.83	80.32 ± 0.49	53.48 ± 4.33	1.51 ± 3.61
F5	50	1	1	55.88 ± 4.87	78.23 ± 0.32	45.47 ± 4.32	1.18 ± 1.58
F6	60	1	1	49.68 ± 5.08	76.51 ± 0.39	39.28 ± 3.79	1.15 ± 3.94
Empty beads	—	1	1	78.01 ± 3.98	—	—	1.42 ± 2.78
F2*∗*	—	—	—	—	67.23 ± 1.03	—	1.45 ± 2.89

*∗*After stability test.

Each observation is the mean values ± SD.

The formulations were hardened by 2% (w/v) of calcium chloride solution.

**Table 2:** Chemical constituents of *O. gratissimum* oil.

Peak	Chemical constituents	Retention Time	RI determined^a^	RI literature^b^		Area (%)	
		(min)			A	B	C
1	Octen-3-ol<1->	8.01	980	979	0.19	0.18	0.18
2	Myrcene	8.4	992	990	0.27	0.28	0.31
3	Ocimene<(Z)-beta->	10.08	1038	1037	14.95	13.88	13.51
4	Ocimene<(E)-beta->	10.47	1049	1050	0.70	0.69	0.77
5	Linalool	12.52	1095	1096	0.63	0.62	0.59
6	Eugenol	23.41	1357	1359	67.38	68.41	68.57
7	Copaene<alpha->	24.16	1376	1376	0.83	0.80	0.79
8	Bourbonene<beta->	24.53	1384	1388	0.09	0.07	0.05
9	Cubebene<beta>	24.76	1386	1388	0.30	0.38	0.31
10	Caryophyllene(E-)	25.96	1417	1419	1.32	1.27	1.29
11	Bergamotene<alpha-trans->	26.6	1436	1434	3.16	3.11	3.09
12	Humulene<alpha>	27.33	1454	1454	0.10	0.16	0.19
13	Germacrene D	28.43	1482	1485	5.25	5.46	5.20
14	Farnesene<(Z,E)-alpha->	28.94	1494	—	4.22	3.91	4.20
15	Farnesene<(E,E)-alpha->	29.46	1509	1505	0.21	0.29	0.31
16	Cadinene<delta>	30.08	1525	1523	0.40	0.46	0.38

A: before entrapping; B: after entrapping; C: entrapped oil after stability test.

^a^Retention indices (RI) calculated from retention times in relation to those of a series of C_8_-C_20_ n-alkanes on a 30m BP-5 capillary column.

^b^Values taken from Adams [37].

## Data Availability

The data used to support the findings of this study are available from the corresponding author upon request.
